# CHARON: An Imaging-Based Diagnostic Algorithm to Navigate Through the Sea of Hereditary Degenerative Ataxias

**DOI:** 10.1007/s12311-024-01677-y

**Published:** 2024-03-04

**Authors:** Alessandra Scaravilli, Mario Tranfa, Giuseppe Pontillo, Bernard Brais, Giovanna De Michele, Roberta La Piana, Francesco Saccà, Filippo Maria Santorelli, Matthis Synofzik, Arturo Brunetti, Sirio Cocozza

**Affiliations:** 1 Department of Advanced Biomedical Sciences, University of Naples “Federico II”, Naples, Italy; 2 Department of Neurology and Neurosurgery, Montreal Neurological Hospital and Institute, Montreal, Canada; 3 Department of Neurosciences and Reproductive and Odontostomatological Sciences, University of Naples “Federico II”, Naples, Italy; 4 Department of Molecular Medicine, IRCCS Stella Maris Foundation, Pisa, Italy; 5 German Center for Neurodegenerative Diseases (DZNE), Tübingen, Germany; 6 Division Translational Genomics of Neurodegenerative Diseases, Center for Neurology and Hertie Institute for Clinical Brain Research, University of Tübingen, Tübingen, Germany

**Keywords:** Ataxia, Neuroimaging, Magnetic Resonance Imaging, Algorithm

## Abstract

The complexity in diagnosing hereditary degenerative ataxias lies not only in their rarity, but also in the variety of different genetic conditions that can determine sometimes similar and overlapping clinical findings. In this light, Magnetic Resonance Imaging (MRI) plays a key role in the evaluation of these conditions, being a fundamental diagnostic tool needed not only to exclude other causes determining the observed clinical phenotype, but also to proper guide to an adequate genetic testing. Here, we propose an MRI-based diagnostic algorithm named CHARON (Characterization of Hereditary Ataxias Relying On Neuroimaging), to help in disentangling among the numerous, and apparently very similar, hereditary degenerative ataxias. Being conceived from a neuroradiological standpoint, it is based primarily on an accurate evaluation of the observed MRI findings, with the first and most important being the pattern of cerebellar atrophy. Along with the evaluation of the presence, or absence, of additional signal changes and/or supratentorial involvement, CHARON allows for the identification of a small groups of ataxias sharing similar imaging features. The integration of additional MRI findings, demographic, clinical and laboratory data allow then for the identification of typical, and in some cases pathognomonic, phenotypes of hereditary ataxias.

## Introduction

Hereditary degenerative ataxias are a large group of heterogeneous diseases, characterized by a progressive onset of uncoordinated gait, usually along with poor eye-hand coordination and dysarthria [1]. These conditions, often presenting with overlap between the clinical phenotypes, can manifest with a pure cerebellar phenotype or as a combination of cerebellar symptoms and extracerebellar features [2].

The complexity in diagnosing hereditary ataxias lies not only in their rarity, but also in the variety of different genetic conditions that can determine them, inherited in an autosomal recessive, autosomal dominant or X-linked manner, or as a part of a mitochondrial genetic syndrome [1]. In the context of this heterogeneity, Magnetic Resonance Imaging (MRI) plays a key role in the evaluation of these conditions, being crucial in the differential diagnosis process not only to exclude other underlying conditions possibly explaining the clinical phenotype, but also in the determination of the pattern of cerebellar involvement. From a neuroradiological standpoint, atrophy of the cerebellum is often the common denominator, and can represent sometimes the only imaging change detectable with conventional imaging in many of these conditions [3–20]. Indeed, in many cases the neuroradiological evaluation of these patients is primarily focused on the detection of this feature, often reported as a vague “cerebellar atrophy” and without a specific evaluation, detection and reporting of the possible selective involvement of the main structures of the cerebellum (e.g. vermis, hemispheres, anterior lobe, posterior lobe, etc.), of the other infratentorial structures (e.g. medulla oblongata, pons, cerebellar peduncles, etc.), or their combination.

MRI usually does not lead to the identification of a specific hereditary ataxia and the definitive diagnosis of these conditions is suggested by the combination of family history, clinical symptoms, neuroimaging and laboratory findings and achieved via molecular genetic testing [13]. In particular, in the last years the growing implementation in clinical practice of Next-Generation Sequencing (NGS) methods has allowed for the identification of more than 200 primary ataxia-associated genes [21, 22]. Although efforts have been made to standardize NGS data sharing and analysis in hereditary ataxias [23], these methods still remain available to relatively few tertiary care centers [24] with costs unlikely to be sustained in most low income Countries. For these reasons, a proper evaluation of a widely accessible and less expensive diagnostic methods such as MRI still represents a fundamental diagnostic tool needed to guide genetic tests to a more appropriate and relatively limited range of genetic forms of ataxias.

Evaluation of MRI alone is not sufficient in differentiating among all these different conditions, because of the high degree of heterogeneity within the same genetic forms [13] but the association of MRI with clinical symptoms and laboratory parameters can guide the diagnosis towards a smaller group of conditions, or in some cases even a specific condition.

## The CHARON Algorithm

Here, we propose a diagnostic algorithm named CHARON (Characterization of Hereditary Ataxias Relying On Neuroimaging), conceived from a neuroradiological standpoint, based on the available knowledge of conventional MRI findings [13] to help in disentangling among the most common hereditary degenerative ataxias. It is based primarily on an accurate evaluation of the MRI findings, with the first and most important being the pattern of cerebellar atrophy, distinguishing between genetic forms of ataxias characterized by the presence of a global cerebellar atrophy from conditions presenting with unremarkable cerebellar volume changes, or atrophy limited to specific cerebellar regions. Neuroradiologists should then evaluate the possible presence (or absence) of concomitant volumetric changes in the infratentorial compartment (e.g., pons, cerebellar peduncles, or medulla oblongata), as well as possible other brain signal changes (e.g., bilateral hyperintensity of the Dentate Nuclei – DN –, or the “Hot Cross Bun” – HCB – sign). Once identified a “cluster” of conditions sharing these similar imaging features, it should be then researched in the first place the presence of additional specific neuroradiological findings (e.g., alterations of the supratentorial compartment both in terms of atrophy and/or signal changes) that, where available, could further help in distinguish between some of these conditions. At this point, the integration of demographic, clinical and laboratory data should allow for the identification of typical, and in some cases pathognomonic, phenotypes of hereditary ataxias.

**Fig. 1:**
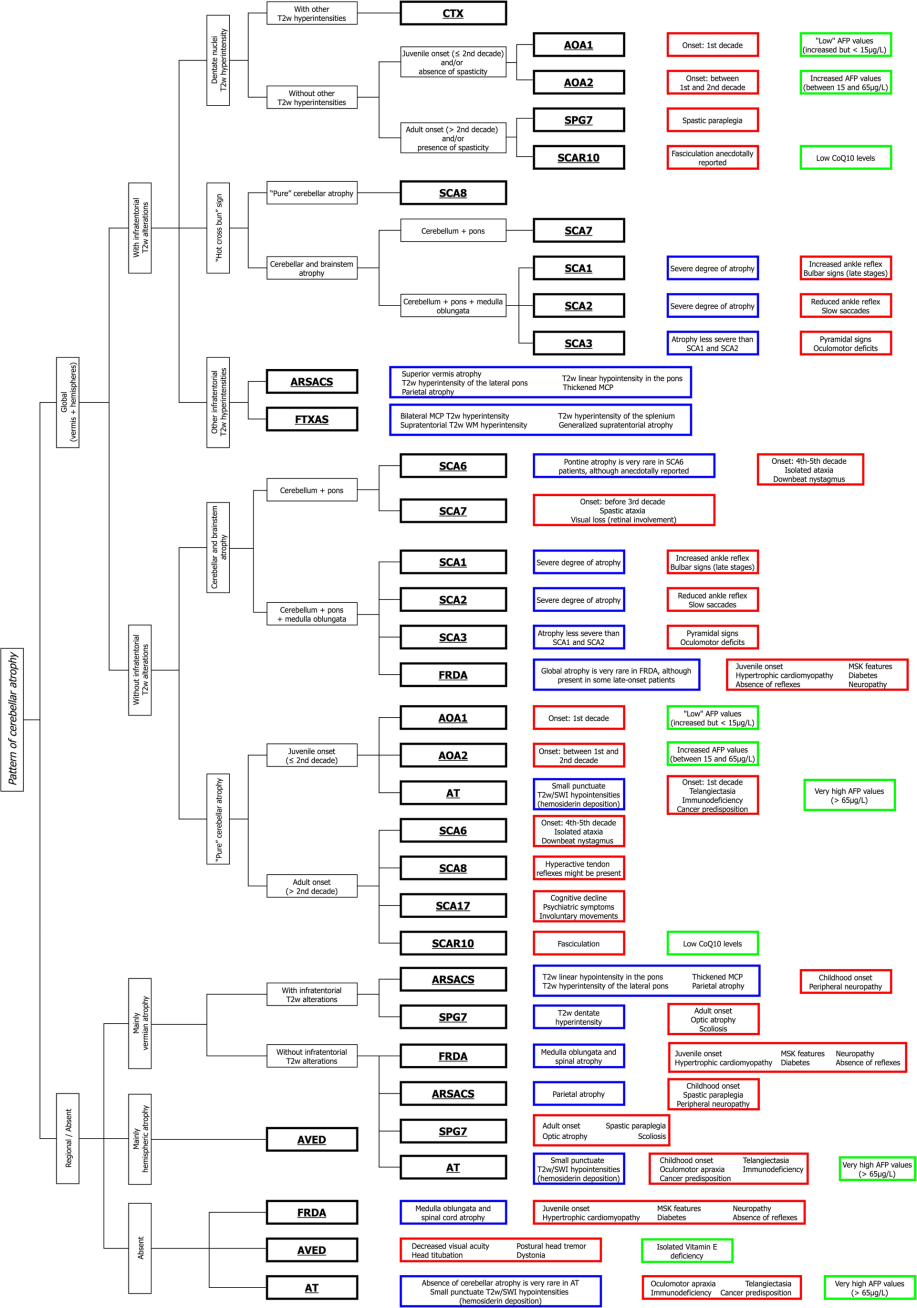
**The CHARON algorithm.** The different ataxias are marked in bold. The blue boxes indicate the additional neuroradiological findings characteristic for each entity, while typical clinical and laboratory data are shown in the red and green boxes, respectively. CHARON: Characterization of Hereditary Ataxias Relying On Neuroimaging; CTX: Cerebrotendinous Xanthomatosis; AOA: Ataxia with Oculomotor Apraxia; SPG7: Spastic Paraplegia Type 7; SCAR10: Autosomal Recessive Spinocerebellar Ataxia Type 10; SCA: Spinocerebellar Ataxia; ARSACS: Autosomal Recessive Spastic Ataxia of Charlevoix-Saguenay; FXTAS: Fragile X-associated Tremor/Ataxia Syndrome; FRDA: Friedreich’s Ataxia; AT: Ataxia-Telangiectasia; AVED: Ataxia with isolated Vitamin E Deficiency; AFP: Alpha-Fetoprotein; CoQ10: Coenzyme Q10

The CHARON algorithm is shown in Fig. 1, with a brief description here following.

**Fig. 2:**
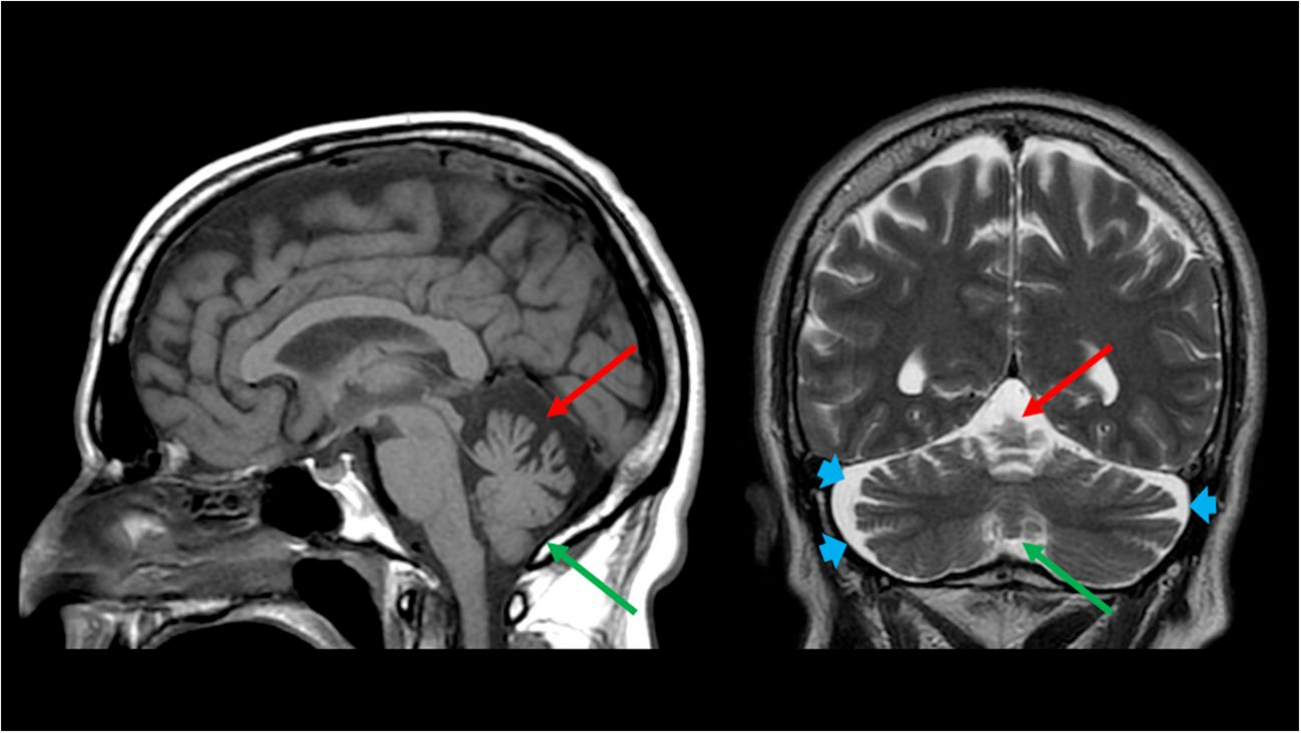
**A pattern of “pure” cerebellar atrophy.** Sagittal T1-weighted (*left*) and coronal T2-weighted (*right*) images of a 54-year-old male Spinocerebellar Ataxia Type 6 patient showing a pattern of “pure” cerebellar atrophy, with a concomitant involvement of the superior vermis (*red arrows*), and the cerebellar hemispheres (depicted by the enlargement of the cerebellar fissures with corresponding increase in CSF spaces—*blue arrowheads*), and a less significant although present volume loss of the inferior vermis (*green arrows*), with a relative sparing of the remaining brain structures

**Fig. 3:**
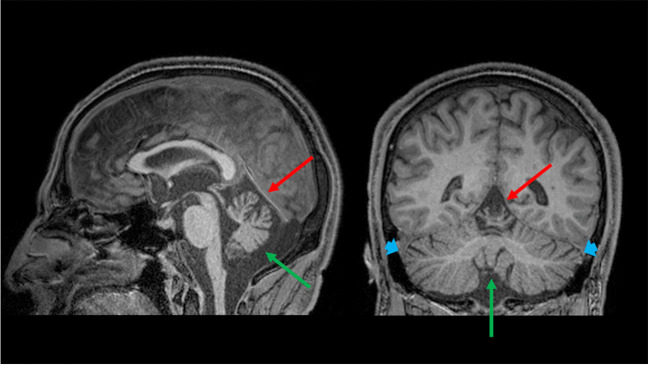
**A pattern of selective cerebellar atrophy.** Sagittal (*left*) and coronal (*right*) multiplanar reconstruction of a T1-weighted volumetric sequence of a 43-year-old male Autosomal Recessive Spastic Ataxia of Charlevoix-Saguenay patient showing a selective atrophy of the superior vermis (*red arrows*), with the remaining cerebellar structures (i.e., inferior vermis—*green arrows*—and cerebellar hemispheres—*blue arrows*) that appear unremarkable

As previously discussed, the first MRI feature that need to be evaluated is represented by the pattern of cerebellar atrophy, that can therefore identify two major classes of hereditary ataxias: those with a “pure” cerebellar atrophy involving both the vermis and the hemispheres (Group A) (Fig. 2) or those without cerebellar atrophy or showing a selective involvement of either the vermis or the hemispheres (Group B) (Fig. 3).

## Ataxias with Global Cerebellar Atrophy – Group A

**Fig. 4:**
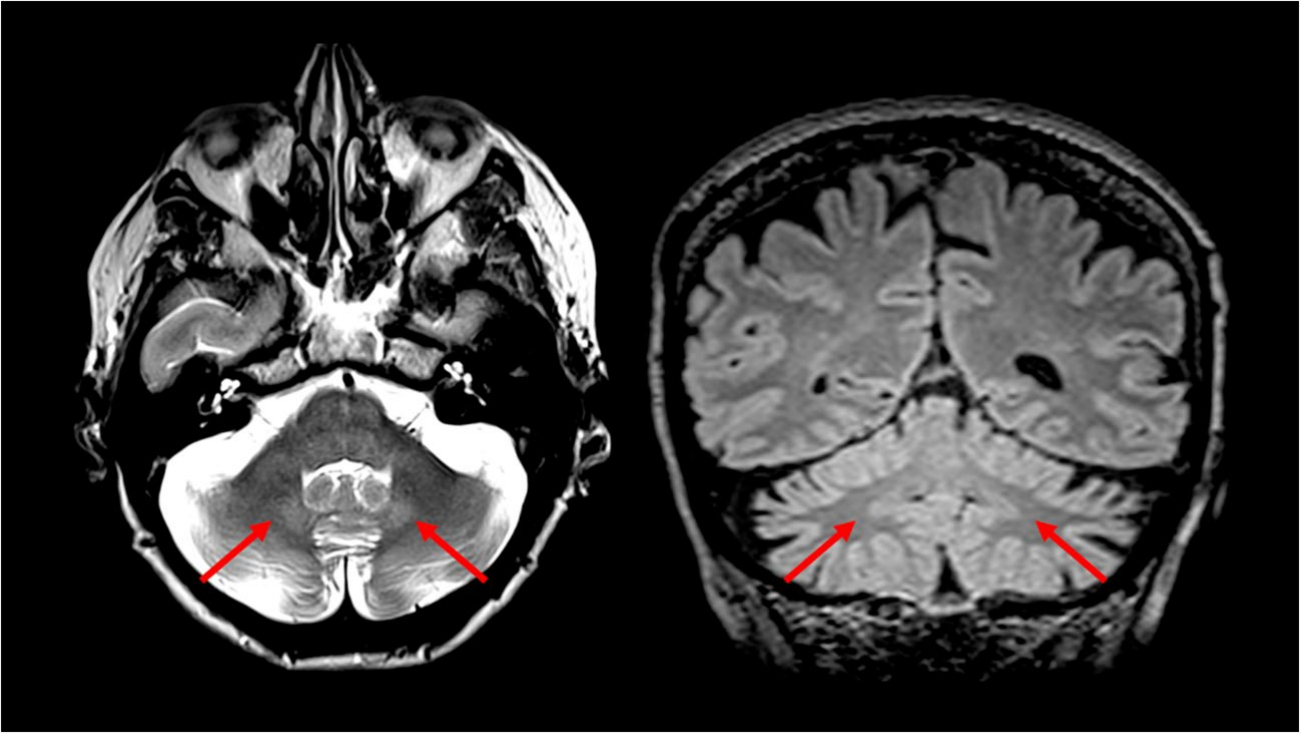
**An example of Dentate Nuclei T2w hyperintensity.** Axial T2-weighted (*left*) and coronal multiplanar reconstruction of a volumetric Fluid Attenuated Inversion Recovery sequence (*right*) of a 70-year-old female Spastic Paraplegia Type 7 patient showing a bilateral T2-weighted hyperintensities of the Dentate Nuclei (*red arrows*)

With reference to Group A, this still wide group of conditions can be further divided in two branches by evaluating the additional presence or absence of infratentorial T2-weighted changes. In particular, if any infratentorial T2-weighted changes are found, these can be divided in three major patterns of signal changes: the presence of a bilateral hyperintensity of the DN, the presence of a HCB sign, and other signal changes affecting the infratentorial compartment. The presence of a bilateral DN T2-weighted involvement (Fig. 4) can direct the diagnosis toward a relatively smaller group of conditions, which in terms of prevalence mostly should suggest Cerebrotendinous Xanthomatosis (CTX), Ataxia with Oculomotor Apraxia Type 1 (AOA1) and 2 (AOA2), Spastic Paraplegia Type 7 (SPG7), and the autosomal recessive Spinocerebellar Ataxia Type 10 (SCAR10) [4, 5, 8, 10, 25, 26]. In the case of the presence of associated signal changes (i.e., a low signal in Susceptibility Weighted Imaging -SWI- surrounding the DN) a diagnosis of CTX can be suggested [8], while the evaluation of the age of onset can help distinguishing between AOA Type 1 and 2 (usually presenting with an onset before the 2nd decade) [27] and SPG7 or SCAR10 (usually presenting with an onset after the 2nd decade) [5, 9, 28]. In the first case, the presence of Alpha-Fetoprotein (AFP) levels below 15 μg/L can orient the diagnosis towards AOA1 rather than AOA2 [29], while the occurrence of spastic paraplegia should suggest SPG7 above SCAR10 [30], that furthermore typically shows low Coenzyme Q10 (CoQ10) values [9].

**Fig. 5:**
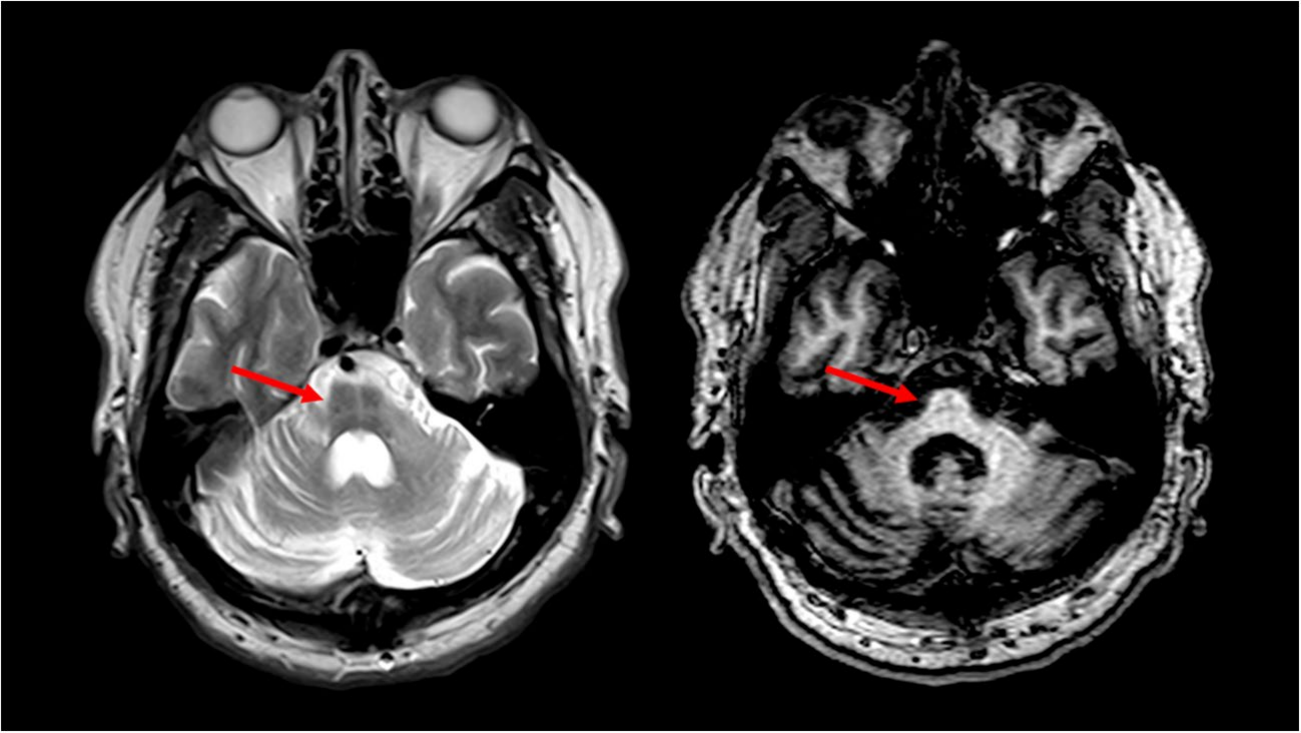
**An example of a “Hot Cross Bun” sign.** Axial T2-weighted (*left*) and axial multiplanar reconstruction of a T1-weighted volumetric sequence (*right*) of a 59-year-old male Spinocerebellar Ataxia Type 2 patient showing an hyperintensity of transverse pontine fibers and median pontine raphe (*red arrows in left*), also visible as a T1-weighted mild hypointensity (*red arrows in right*), representing the so-called “Hot Cross Bun” sign

On the other hand, the presence of a positive HCB sign (Fig. 5) has been reported in several hereditary ataxias, including Spinocerebellar Ataxia (SCA) Type 1, 2, 3, 7 and 8 [18, 31–33]. In this case, the presence of a positive HCB sign without brainstem involvement might suggest the observation of a SCA8 patient, while its presence coupled with a selective pontine atrophy can be found in SCA7 [16, 17]. Finally, the combination of this sign with an atrophy of cerebellum, the pons and the medulla oblongata should suggest a diagnosis between SCA1, SCA2, SCA3 [14, 15, 18, 31], conditions very hard to distinguish purely from a neuroradiological standpoint (although the degree of atrophy is less prominent in SCA3 compared to SCA1 and 2 [12]). In this case, the clinical evidence of alteration of ankle reflexes, pyramidal signs, or oculomotor deficits, can indicate a more plausible diagnosis [34–36]. Finally, the presence of other T2-weighted changes, such as the evidence of white matter lesions in middle cerebellar peduncles and splenium of the corpus callosum, or at the level of the lateral portion of the pons (also appearing thickened), can help in discriminate between the Fragile X-associated Tremor/Ataxia Syndrome (FXTAS) and Autosomal Recessive Spastic Ataxia of Charlevoix-Saguenay (ARSACS), respectively [11, 20, 37].

In absence of infratentorial T2-weighted detectable changes, neuroradiologists should carefully evaluate the possible brainstem involvement. In the presence of a “pure” cerebellar atrophy pattern, age of disease onset may help in achieving a proper diagnosis. Indeed, a juvenile onset (usually before the 2nd decade) is more typical in AOA1, AOA2, and Ataxia-Telangiectasia (AT) [27, 38], while adult-onset ataxias with this MRI pattern mostly include SCA6, SCA8, SCA17 and SCAR10 [9, 28, 39–41]. The differential diagnosis between AOA1, AOA2 and AT can be achieved by showing the typical SWI changes at the level of the deep with matter in AT [6, 42], while the evaluation of laboratory parameters such as the presence of AFP values can help in the diagnostic process, given that AT patients usually show very high levels (more than 65 μg/L), while AOA1 patients only rarely show values higher than 15 μg/L [29]. On the other hand, specific clinical features can help in discriminate between adult-onset hereditary ataxias with a “pure” cerebellar atrophy without T2-weighted changes, such as the presence of downbeat nystagmus in SCA6 [43, 44], cognitive and psychiatric symptoms in SCA17 [45, 46] or fasciculations in SCAR10 [47].

## Ataxias without Global Cerebellar Atrophy – Group B

The other major group of conditions are those hereditary ataxias showing no cerebellar atrophy or only a selective involvement of a specific part of the cerebellum (Group B). In case of ataxia and preserved cerebellar volumes, Friedreich’s Ataxia (FRDA) is by far the most common in term of prevalence [48], being usually characterized by a normal brain MRI scan, with only a mild atrophy of the medulla oblongata and cervical spinal cord in some cases [49, 50]. The other main condition characterized by a sparing of the cerebellum is the Ataxia with Vitamin E Deficiency (AVED), in which a differential diagnosis with FRDA can be relatively easily achieved by evaluating indeed Vitamin E levels, reduced in AVED patients and preserved in FRDA [51, 52]. On the other hand, the prominent involvement of the vermis over the cerebellar hemispheres is more challenging, given the higher number of conditions showing this MRI feature. In these cases, as already discussed for conditions of Group A, neuroradiologists should evaluate the possible presence of T2-weighted changes of the infratentorial compartment. Their presence should indicate the possibility of observing either SPG7 or ARSACS [5, 20], with the differential diagnosis between these two diseases that can be achieved via the identification of peculiar MRI (i.e. mostly superior vermian involvement, pontine thickening, and parietal atrophy in ARSACS [37], DN T2-weighted hyperintensity in SPG7 [5]) or clinical (e.g. peripheral neuropathy in ARSACS [53], optic atrophy in SPG7 [54]) findings. It is noteworthy to stress that the absence of T2-weighted changes cannot exclude a possible observation of any these two conditions, as well as the possible occurrence of FRDA or AT. In this case, an accurate evaluation of clinical findings (e.g. the association of diabetes mellitus and skeletal abnormalities in FRDA [48, 55], immunodeficiency in AT [38] or the abovementioned neuropathy and optic atrophy in ARSACS and SPG7 [53, 54]), in combination with peculiar laboratory changes (i.e. elevated AFP levels in AT [56]) can help in achieving a proper diagnosis.

## Limitations and Conclusions

This algorithm clearly has some limitations, with the first being that it has been developed over a finite and relatively small number of hereditary ataxias (namely, those reported with the highest known prevalence to date). Furthermore, it still misses a real-world validation and, most importantly, certainly cannot be used to reach a final and definite diagnosis of ataxia given the unquestionable role of molecular diagnosis.

Nevertheless, CHARON can represent a useful tool for the neuroradiologists, that having available relatively basic clinical and laboratory findings and properly evaluating MRI findings, can identify patterns of brain changes guiding neurologists towards a more appropriate genetic testing of more limited and targeted number of conditions, avoiding long and expensive tests, with the final aim of achieving a correct and fast diagnosis.

## Data Availability

No datasets were generated or analysed during the current study.

## Abbreviations

AFP: Alpha-Fetoprotein
AOA: Ataxia with Oculomotor Apraxia
ARSACS: Autosomal Recessive Spastic Ataxia of Charlevoix-Saguenay
AT: Ataxia-Telangiectasia
AVED: Ataxia with isolated Vitamin E Deficiency
CHARON: Characterization of Hereditary Ataxias Relying On Neuroimaging
CoQ10: Coenzyme Q10
CTX: Cerebrotendinous Xanthomatosis
DN: Dentate Nuclei
FRDA: Friedreich’s Ataxia
FXTAS: Fragile X-associated Tremor/Ataxia Syndrome
SCA: Spinocerebellar Ataxia
SCAR10: Autosomal Recessive Spinocerebellar Ataxia Type 10
SPG7: Spastic Paraplegia Type 7
